# Diphlorethohydroxycarmalol Inhibits Interleukin-6 Production by Regulating NF-κB, STAT5 and SOCS1 in Lipopolysaccharide-Stimulated RAW264.7 Cells

**DOI:** 10.3390/md13042141

**Published:** 2015-04-13

**Authors:** Na-Jin Kang, Sang-Chul Han, Gyeoung-Jin Kang, Dong-Hwan Koo, Young-Sang Koh, Jin-Won Hyun, Nam-Ho Lee, Mi-Hee Ko, Hee-Kyoung Kang, Eun-Sook Yoo

**Affiliations:** 1 Department of Biomedicine & Drug Development, Jeju National University, Jeju 690-756, Korea; E-Mails: najin0423@naver.com (N.-J.K.); piofpe87@gmail.com (D.-H.K.); yskoh7@jejunu.ac.kr (Y.-S.K.); jinwonh@jejunu.ac.kr (J.-W.H.); pharmkhk@jejunu.ac.kr (H.-K.K.); 2 Department of Medicine, Jeju National University School of Medicine, Jeju 690-756, Korea; E-Mails: hanschh@naver.com (S.-C.H.); izar0824@nate.com (G.-J.K.); 3 Department of Chemistry, School of Natural Science, Jeju National University, Jeju 690-756, Korea; E-Mail: namho@jejunu.ac.kr; 4 Jeju Biodiversity Research Institute (JBRI), JejuTechnopark (JTP), Jeju 690-787, Korea; E-Mail: miheeko@jejutp.or.kr

**Keywords:** diphlorethohydroxycarmalol (DPHC), IL-6, NF-κB, Jak-STAT, SOCS, LPS, inflammation

## Abstract

Diphlorethohydroxycarmalol (DPHC) is a phlorotannin compound isolated from *Ishige okamuarae*, a brown alga. This study was conducted to investigate the anti-inflammatory effect and action mechanism of DPHC in lipopolysaccharide (LPS)-stimulated RAW 264.7 macrophages. We found that DPHC strongly reduces the production of interleukin 6 (IL-6), but not that of tumor necrosis factor-alpha (TNF-α) induced by LPS. DPHC (12.5 and 100 μM) suppressed the phosphorylation and the nuclear translocation of NF-kappaB (NF-κB), a central signaling molecule in the inflammation process induced by LPS. The suppressor of cytokine signaling 1 (SOCS1) is a negative feedback regulator of Janus kinase (Jak)-signal transducer and activator of transcription (STAT) signaling. In this study, DPHC inhibited STAT5 expression and upregulated that of SOCS1 at a concentration of 100 μM. Furthermore, *N*-tosyl-l-phenylalanine chloromethyl ketone (TPCK) (a specific NF-κB inhibitor) and JI (a specific Jak2 inhibitor) reduced the production of IL-6, but not that of tumor necrosis factor-alpha (TNF-α) in LPS-stimulated RAW 264.7 macrophages. These findings demonstrate that DPHC inhibits IL-6 production via the downregulation of NF-κB and Jak2-STAT5 pathway and upregulation of SOCS1.

## 1. Introduction

Inflammation is an important body defense mechanism against pathogens and diverse external stimuli. Numerous endogenous inflammatory mediators, including cytokines, chemokines, prostaglandins and nitric oxide (NO), are present in the body. Among these mediators, interleukin (IL)-6 and tumor necrosis factor-α (TNF-α) are representative pro-inflammatory cytokines, and their overproduction exacerbate various inflammatory states, including sepsis and rheumatoid arthritis [1,2]. These cytokines are generated via sequential molecular signaling events after stimulation of a type of toll-like receptor (TLR) on the surface of macrophages by pathogens or microbial products [3,4]. TLR4, a member of the TLRs family, recognizes lipopolysaccharide (LPS), a wall component of Gram-negative bacteria, and this recognition leads to inflammation [5]. LPS, an inducer of innate immune response, binds to a myeloid differentiation factor-2 (MD-2)/TLR4 complex, initiates the downstream signaling pathway and releases various inflammatory chemokines and cytokines, including IL-6, inducible nitric oxide synthase (iNOS) and TNF-α [6,7,8]. In the downstream signaling pathway, intracellular adaptor proteins lead to two distinct signaling pathways, namely the MyD88-dependent and MyD88-independent signaling pathway. The MyD88-dependent pathway includes mitogen-activated protein kinases (MAPK) (c-Jun N-terminal kinase (JNK), p38 and extracellular-signal related kinase (ERK)) and the NF-kappaB (NF-κB; p50/p65) complex. Meanwhile, the MyD88-independent signaling pathway produces type 1 interferons (IFNs). These types 1 IFNs regulate transcription factors with signal transducers and activators of transcription 1 (STAT1) [9]. NF-κB is one of the most principal transcription factors and a central inflammatory mediator, and this molecule is involved in the gene induction of cellular proliferation and inflammation [10]. When a stimulus is applied, NF-κB is activated through the activation of the I kappa B-kinase (IKK) complex, which phosphorylates I kappa B-alpha (IκB-α) and p50/p65. IκB-α is then produced because of proteasomal degradation, and NF-κB mainly exists as a heterodimer, including subunits p50/p65 of the Rel family. A free p50/p65 complex translocates from cytosol to nucleus and finally controls the promotor region of target genes, which induces various pro-inflammatory factors [11,12].

STATs are a family of nuclear proteins that mediate the action of several cytokines, such as ILs, IFNs and others. The Janus kinase (Jak) pathway is triggered by various ligands, including cytokines, and it activates immune and inflammatory responses, as well as other cellular events [13,14]. Jak2 of the Janus kinase family is predominantly expressed in macrophages and is an important modulator of immune responses [15,16]. It has been confirmed that Jak2 is required for the activation of STAT signaling following an interaction of cytokine/IFN receptors with their ligands [17]. The suppressor of cytokine signaling 1 (SOCS1) was known as one of the intracellular negative-feedback molecules that inhibits Jak-STAT activation initiated by various cytokines, including IFN-gamma, IL-6, IL-4 and IL-12 [18,19,20]. Kimura *et al.* reported that Jak2 and Stat5 are directly activated by LPS, whereas SOCS1 inhibits LPS-induced Jak2 and Stat5 activation in macrophages [21].

Various marine bio-resources have recently been explored because of the investigations of active components in pharmaceutical and functional food areas [22,23]. Diphlorethohydroxycarmalol (DPHC) was discovered during the determination of anti-inflammatory materials from marine plants living in the Jeju coastal area. DPHC has been isolated from a brown alga called *Ishige okamuarae*. Few studies have been conducted on the useful biological activities of DPHC. This compound exhibited an anti-diabetic effect that alleviated increased postprandial blood glucose levels in diabetic mice and induced factors of high glucose-induced oxidative stress in human umbilical vein endothelial cells [24,25]. However, little information is available regarding the anti-inflammatory activity of DPHC.

In the present study, we investigated the activity and action mechanism of DPHC on the production of IL-6, a pivotal cytokine of the inflammatory process in LPS-stimulated RAW264.7 cells.

## 2. Results and Discussion

### 2.1. DPHC Selectively Inhibits LPS-Induced IL-6 Production in RAW264.7 Cells

**Figure 1:**
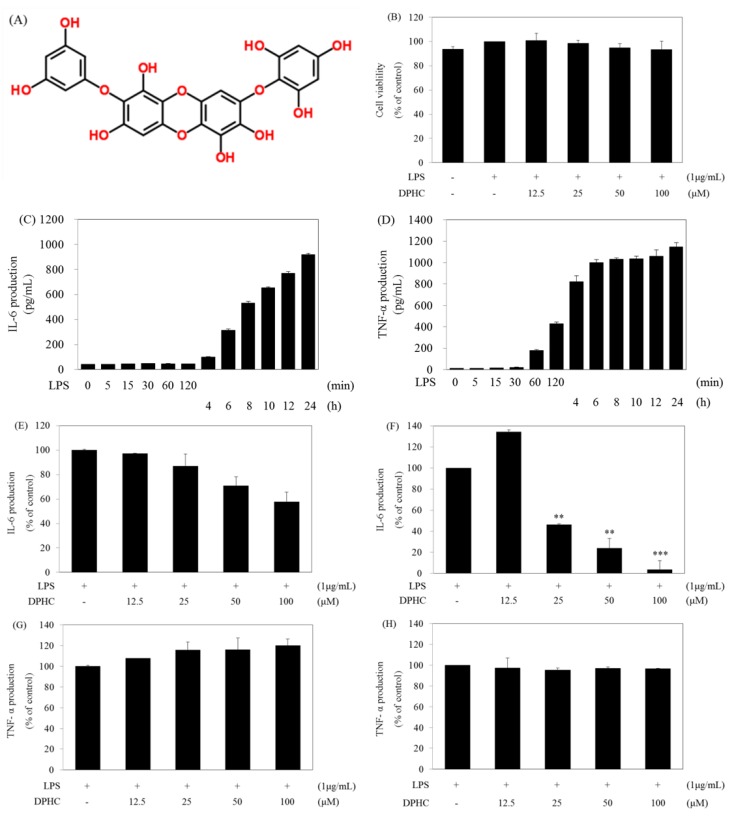
Effect of diphlorethohydroxycarmalol (DPHC) on the production of IL-6 and TNF-α in LPS-stimulated RAW264.7 cells. (**A**) Structure of DPHC; (**B**) Cells (1.5 × 10^5^ cells/mL) were pre-incubated for 18 h and then treated with LPS in the presence or absence of DPHC for 24 h. Cell viability was determined by the WST assay; (**C**,**D**) Cells (1.5 × 10^5^ cells/mL) were treated with LPS for 24 h. The amounts of IL-6 or TNF-α were measured from the culture supernatants at the different incubation time of LPS; (**E**–**H**) The cells were stimulated with LPS in the presence or absence of DPHC for 6 (Figure 1**E**,**G**) or 24 h (Figure 1F,H). The amounts of IL-6 or TNF-α were measured from the culture supernatants by ELISA. Data are the mean ± SD of three independent experiments. * *p* < 0.05, ** *p* < 0.01 and *** *p* < 0.001 *vs.* DPHC-untreated cells in the presence of LPS.

IL-6 and TNF-α are representative pro-inflammatory cytokines generated by external stimuli in macrophages. To assess DPHC for its anti-inflammatory effect, we investigated the inhibitory activity of DPHC on LPS-induced IL-6 and TNF-α production, important pro-inflammatory cytokines, in RAW264.7 cells. In order to confirm the effect of DPHC, we simultaneously determined cell viability at various concentrations of DPHC by the water soluble tetrazolium salts (WST) assay. As shown in Figure 1B, DPHC is no cytotoxic at the tested concentrations (Figure 1B). In order to find the time profile of IL-6 and TNF-α production, we measured the amount of IL-6 and TNF-α in culture supernatants at the indicated time points after LPS treatment. After LPS treatment, TNF-α production was induced after 1 h and continuously increased up to 4 h and then plateaued (Figure 1D), whereas the IL-6 production continuously increased from 6 h (312.4 pg/mL ± 10.2) up to 24 h (918.3 pg/mL) (Figure 1C), and TNF-α was produced earlier than IL-6. These results of the production of IL-6 and TNF-α according to the LPS treatment times are correlated with the kinetics of mRNA expression of inflammatory mediators reported in our previous article [26]. Next, we examined the effect of DPHC on the production of IL-6 and TNF-α induced by LPS. DPHC inhibited the production of IL-6 at 6 h (Figure 1E) and 24 h (Figure 1F) in a dose-dependent manner. Especially, the powerful effect of DPHC after 24 h of incubation is shown in Figure 1F. However, DPHC had no effect on the production of TNF-α at both 6 h (Figure 1G) and 24 h (Figure 1H) of incubation. These results provide a possibility that there will be a different mechanism in the production of IL-6 and TNF-α in LPS-stimulated macrophages. Most of the bio-active compounds used in our previous studies inhibited the production of IL-6 and TNF-α in LPS-stimulated macrophages [27]. Therefore, we tried to find the point that DPHC selectively inhibits IL-6 production and that will be a different mechanism in the production of IL-6 and TNF-α in LPS-stimulated murine macrophages.

### 2.2. DPHC Inhibits the Activation and Nuclear Translocation of NF-κB in LPS-Stimulated RAW264.7 Cells

**Figure 2:**
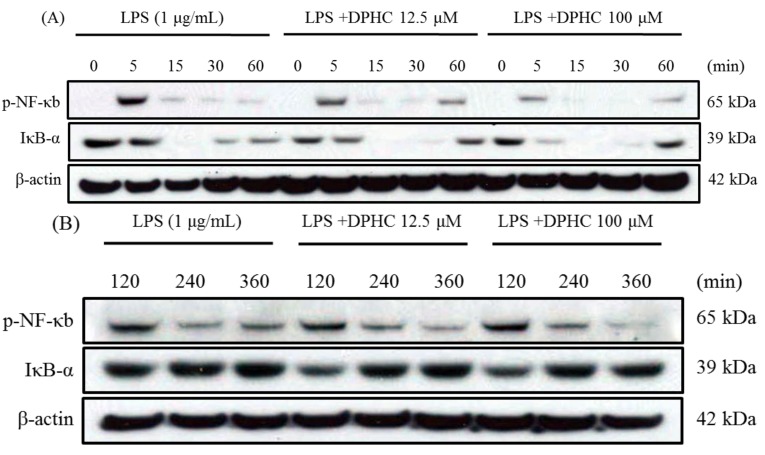

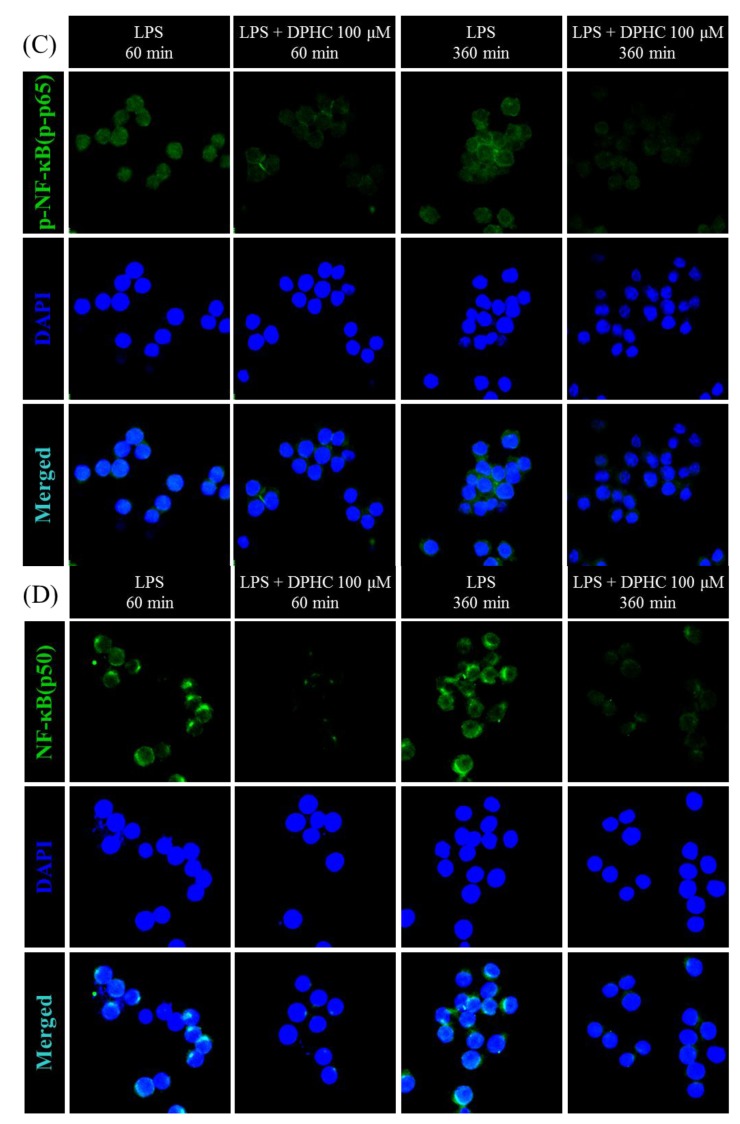
Effect of DPHC on the NF-κB pathway in LPS-stimulated RAW 264.7 cells. (**A**,**B**) Cells (7.5 × 10^5^ cells/mL) were stimulated with LPS in the presence or absence of DPHC. Whole cell lysates were obtained at the indicated time points. NF-κB phosphorylation and IκB-α degradation were assessed by Western blotting from whole cell lysates; (**C**,**D**) Cells (2.0 × 10^5^ cells/mL) were stimulated with LPS in the presence or absence of DPHC for the indicated time intervals. The images were acquired at constant photomultiplier (PMT), gain, offset, magnification (40× oil immersion objectives with a zoom factor of 3.0) and resolution.

LPS presentation on monocytes and macrophages may mediate the activation of the NF-κB pathway, leading to the generation of pro-inflammatory cytokines, including IL-6, iNOS, TNF-α. There are many anti-inflammatory compounds isolated from plants inhibiting the production of inflammatory mediators by regulating NF-κB. Lycopene, a red carotenoid pigment occurring in tomatoes and several other ripe fruits, inhibited the LPS-induced production of NO and IL-6 by suppressing the activation of ERK, p38 and NF-κB in LPS-stimulated RAW264.7 cells [28]. Since phosphorylation of NF-κB-p65 is a crucial step in the function of NF-κB-p65, we determined the phosphorylation of nuclear NF-κB-p65 with and without DPHC treatment by Western blot analysis. DPHC inhibited LPS-induced phosphorylation of NF-κB-p65 at a concentration of 12.5 and 100 μM, and this inhibitory effect has been shown at 5, 60 and 360 min after DPHC treatment (Figure 2A,B). To determine whether this inhibition is accompanied by the degradation of IκB-α, we determined the cytoplasmic levels of IκB-α. As shown in Figure 2A,B, the band intensity of IκB-α decreased at 5 min and returned to basal levels at 60 min in the presence of DPHC (100 μM) after LPS stimulation (Figure 2A). Next, we examined the nuclear translocation of NF-κB (phospho-p65, p50) using confocal laser scanning microscopy. DPHC (100 μM) strongly inhibited LPS-induced nuclear translocation of NF-κB (p65, p50) at 60 and 360 min (Figure 2C,D). These results suggest that the inhibitory effect of DPHC on the production of IL-6 occurs through inhibiting the activation and nuclear translocation of NF-κB.

### 2.3. DPHC Does Not Affect the MAPK Pathway in LPS-Stimulated RAW264.7 Cells

**Figure 3:**
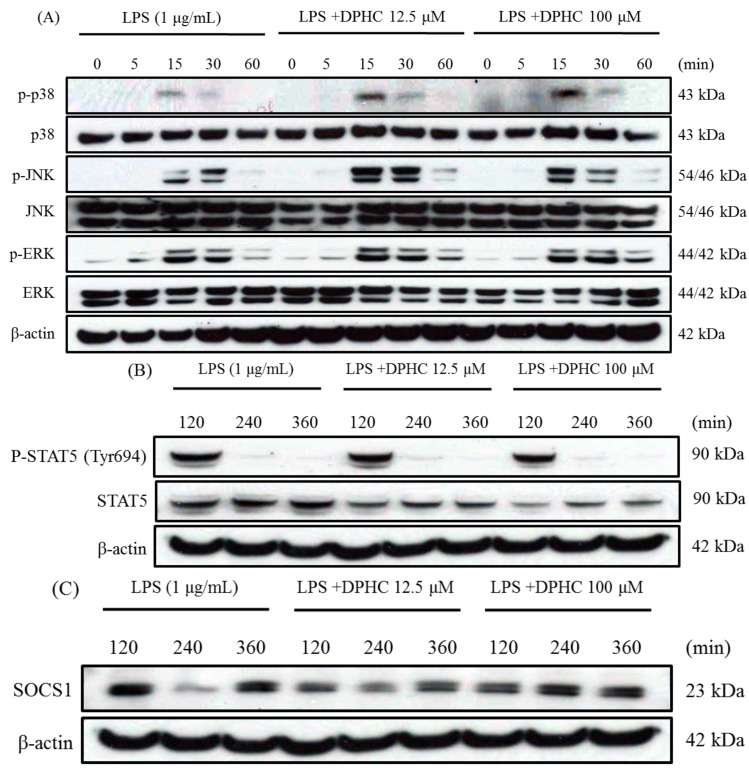
Effect of DPHC on the MAPK pathway, STAT5 and SOCS1 in LPS-stimulated RAW 264.7 cells. (**A**) Cells (7.5 × 10^5^ cells/mL) were stimulated with LPS in the presence or absence of DPHC. Whole cell lysates were obtained at the indicated time points. The phosphorylations of p38, JNK and ERK were assessed by Western blotting from whole cell lysates; (**B**) Cells (7.5 × 10^5^ cells/mL) were stimulated with LPS in the presence or absence of DPHC. Whole cell lysates were obtained at the indicated time points. The STAT5 level was assessed by Western blotting from whole cell lysates. (**C**) Cells (7.5 × 10^5^ cells/mL) were stimulated with LPS in the presence or absence of DPHC. Whole cell lysates were obtained at the indicated time points. The SOCS1 level from whole cell lysates was assessed by Western blotting.

Stimulation of TLR4 by LPS triggers the activation of the MAPK pathway and results in the production of pro-inflammatory cytokines. There are many anti-inflammatory compounds isolated from plants inhibiting the production of inflammatory mediators by regulating the NF-κB, MAPKs and/or Jak-Stat pathways. Theaflavin, a major polyphenol in black tea, suppressed LPS-induced IL-6, MCP-1 and ICAM-1 expression via blockade of the NF-κB and MAPK pathways in bone marrow-derived macrophages [29]. Thus, we examined the effect of DPHC on LPS-induced MAPK activation by Western blotting at various times after LPS treatment. As the results, DPHC (12.5 and 100 μM) did not inhibit the phosphorylation of three MAPKs (p38, JNK and ERK) induced by LPS treatment (Figure 3A). On the contrary, DPHC weakly increased the phosphorylation of p-38 and JNK at the times of 15 and 30 min. Therefore, DPHC did not suppress the phosphorylation of the three MAPKs induced by LPS treatment. In the present study, the anti-inflammatory effect of DPHC was associated with the NF-κB pathway, rather than the MAPK pathway.

### 2.4. DPHC Inhibits STAT5 and SOCS1 in LPS-Stimulated RAW264.7 Cells

STATs are a family of nuclear proteins mediating the action of a number of cytokines, such as ILs, IFNs, and others. Especially, the Jak2-STAT5 pathway is known to be involved in LPS-induced IL-6 production. SOCS1 is a negative feedback regulator of JaK-STAT signaling. A recent study reports that SOCS1, an intracellular negative-feedback molecule, selectively inhibits LPS-induced IL-6 production, but not that of TNF-α [21]. They found that LPS directly activates Jak2 and Stat5, whereas SOCS1 inhibits LPS-induced Jak2 and Stat 5. These authors also demonstrated that Stat5 associates with p50 and mediates LPS-induced IL-6 production. It has been demonstrated that SOCS1 selectively inhibits IL-6 production via the regulation of the Jak2-STAT5 pathway in LPS-stimulated macrophages [21]. Therefore, it is possible that STAT5 and SOCS1 participate in the inhibition of IL-6 production by DPHC treatment. Our study supports the present experimental result that DPHC strongly inhibited IL-6 production, but not that of TNF-α. Therefore, we investigated whether Jak-STAT pathway and SOCS1 levels correlated with the inhibition of IL-6 production by DPHC treatment. We first examined whether DPHC affects STAT5 activation in LPS-stimulated Raw264.7 cells. The phosphorylation of STAT5 peaked after 120 min of LPS stimulation, and this activation was reduced by treatment of 100 μM of DPHC. In addition, DPHC (100 μM) reduced the total protein levels of Stat5 (Figure 3B). We next measured the SOCS1 (an intracellular negative-feedback molecule) level under the same experiment condition of the STAT5 examination. It was found that the SOCS1 level was increased at 240 and 360 min after treatment of 100 μM of DPHC (Figure 3C). Interestingly, it has not yet been reported that a natural anti-inflammatory compound inhibits only the production of IL-6, not that of TNF-a. Furthermore, the regulation of inflammatory cytokines in response to SOCS1 signaling in LPS-stimulated macrophages has not been well studied.

These results suggest that DPHC inhibits IL-6 production by downregulating STAT5 activation and SOCS1 augmentation. However, it is not clear that the evoked SOCS1 protein by DPHC directly inhibits the Jak2-STAT5 pathway.

**Figure 4:**
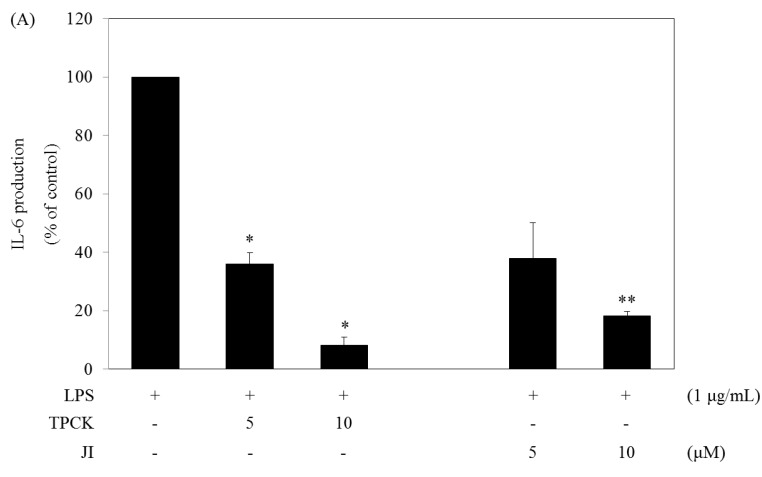

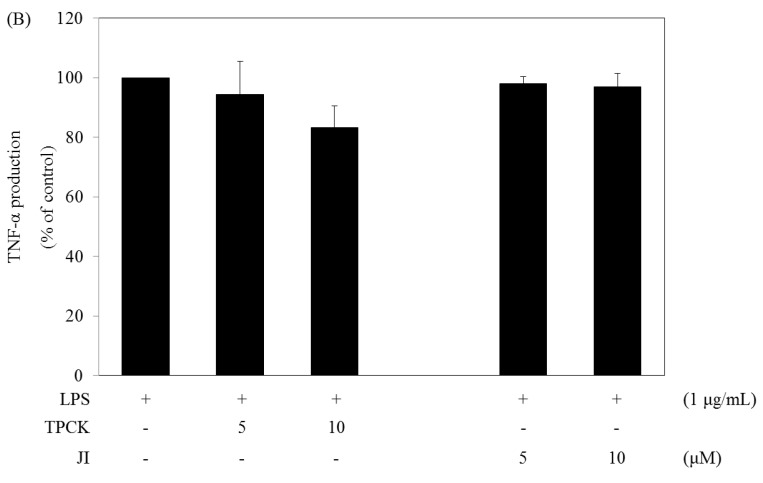
Effects of signaling pathway inhibitors on the production of IL-6 and TNF-α in LPS-stimulated RAW264.7 cells. (**A**,**B**) Cells (1.5 × 10^5^ cells/mL) were treated with LPS in the absence or presence of signaling inhibitors (N-tosyl-L-phenylalanine chloromethyl ketone (TPCK), Jak2 inhibitor (JI)) for 24 h. The IL-6 and TNF-α levels were measured from the culture supernatants by the ELISA method. Data are the mean ± SD of three independent experiments. * *p* < 0.05, ** *p* < 0.01 and *** *p* < 0.001 *vs.* inhibitor-untreated cells in the presence of LPS.

### 2.5. N-Tosyl-L-phenylalanine Chloromethyl Ketone and JI Selectively Reduce the Production of IL-6

The above results show that the NF-κB and Jak2-STAT5 pathways are important in the function of DPHC. To determine whether the NF-κB and Jak2-STAT5 pathways limited to IL-6 production, we examined the cytokine level (IL-6 and TNF-α) after the treatment of two known signaling inhibitors in LPS-stimulated RAW264.7 cells. TPCK (N-tosyl-L-phenylalanine chloromethyl ketone, a specific NF-κB signaling inhibitor) and JI (a specific Jak2 inhibitor) strongly reduced the production of IL-6, but not that of TNF-α induced by LPS (Figure 4).

### 2.6. DPHC Suppresses the Development of Experimental Atopic Dermatitis

**Figure 5:**
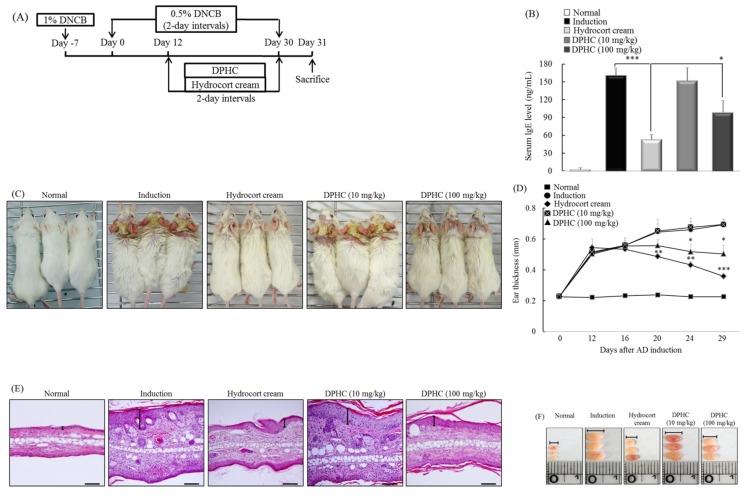
DPHC suppresses experimental atopic dermatitis (AD). (**A**) Mice were challenged with 2,4-dinitrochlorobenzene (DNCB). From Day 12, mice were painted with DPHC (10 and 100 mg/kg) on the ears on every other day; (**B**) The IgE level in serum was measured by ELISA; (**C**) Macroscopic views of the ears (1 ×) and (**D**) ear thickness measured on Days 0, 12, 16, 20, 24 and 29; (**E**) Paraffin-embedded sections of ear tissue stained with H & E. Scale bar = 0.1 mm; (**F**) The lymph nodes (LNs) were photographed to record morphologic changes. (*n* = 10 mice per group). Values represent the mean ± SD. * *p* < 0.05, ** *p* < 0.01 and *** *p* < 0.001 compared with mice in the induction group.

To induce experimental atopic dermatitis (AD), mice were sensitized by applying 1% 2,4-dinitrochlorobenzene (DNCB) to the abdomen. They were then re-sensitized by applying 0.3% DNCB to the ears on every other day for up to 30 days. Starting on Day 12, the mice were painted with hydrocort cream and DPHC (10 and 100 mg/kg) on the ears on every other day (Figure 5A). IgE is an important therapeutic target for AD, as it is the major activator of mast cells, which release histamine [30]. Therefore, we measured the levels of serum IgE in mice with dermatitis. The DPHC-treated group showed significantly reduced levels of IgE (*p* < 0.05) compared with the induction-only group (mice exposed to DNCB, but not having DPHC applied; Figure 5B). The skin lesions associated with AD are characterized by an inflammatory cell infiltrate [31]. Therefore, we next tested whether DPHC reduced the level of inflammatory cell infiltration in the ears of mice with experimental AD. We also examined cutaneous edema as a measure of AD progression. We found that the ear thickness in DPHC-treated mice was reduced at Days 24 and 29 (both *p* < 0.05) compared with that in induction-only mice (Figure 5C,D). We next examined the effect of DPHC on the infiltration of inflammatory cells by hematoxylin and eosin (H&E) staining of ear tissue sections. Epidermal thickness and the degree of inflammatory cell infiltration were significantly lower in the DPHC-treated group than in the induction-only group (Figure 5E). The lymph nodes (LNs) play an important role in cell-mediated immunity by regulating the activity of mature T- and B-cells [32]. Therefore, we examined the morphologic changes in the LN of AD mice. The LNs from mice in the induction-only group were very swollen, whereas those from DPHC mice were smaller and weighed less (Figure 5F).

## 3. Experimental Section

### 3.1. Reagents

LPS (*E. coli* 0111:B4) was purchased from Sigma-Aldrich Chemical Co. (St. Louis, MO, USA). Fetal bovine serum (FBS) and Dulbecco’s Modified Eagle’s Medium (DMEM) were obtained from Invitrogen-GIBCO (Grand Island, NY, USA). Mouse IL-6 Duoset enzyme-linked immunosorbent assay (ELISA) kits were obtained from R&D Systems (St. Louis, MO, USA). MAPKs (anti-phospho-ERK1/2, anti-ERK1/2, anti-phospho-JNK, anti-JNK), the signaling transducer and activator of transcription (STATs; anti-phospho-STAT5 (Tyr694), anti-STAT5) and NF-κB (anti-phospho-p65, anti-p65, anti-IκB-α) and phospho-p38 MAP kinase (Thr180/Tyr182), anti-p38, and anti-SOCS1 were purchased from Cell Signaling Technology (Beverly, MA, USA); NF-κB (anti-p50 (E-10)) was purchased from Santa Biotechnology, Inc. (Santa Cruz, CA, USA); β-actin was obtained from Sigma (St. Louis, MO, USA); and DyLight 488 conjugated Donkey anti-Rabbit antibody was purchased from BioLegend Inc. (San Diego, CA, USA). All other reagents were reagent grade.

### 3.2. Experimental Animals

Female BALB/c mice (7 weeks old) were purchased from Orient Bio (Orient Bio Inc., Seongnam, Korea) and maintained under pathogen-free conditions in the animal facility of Jeju National University. All animal experiments were approved by the Jeju National University Animal Care and Use Committee (2013-0025).

### 3.3. DPHC Isolation

Thalli of *Ishige okamuarae* were collected on Jeju Island, Korea. A voucher specimen has been deposited at the herbarium of Jeju Biodiversity Research Institute (JBR-85). A shade-dried whole plant of *Ishige okamuarae* (800 g) was extracted with 70% aqueous ethanol under stirring (750 rpm) for 2 days at room temperature. The filtrate was concentrated under reduced pressure and freeze-dried to obtain a powder. The filtrate was suspended in distilled water and partitioned with ethyl acetate. The ethyl acetate fraction was chromatographed in a reversed phase silica gel using gradient solvent (H_2_O/MeOH) system to provide four fractions (1–4). Fraction 1 was chromatographed in a Sephadex-LH 20 column (Pharmacia, Stockholm, Sweden) using CHCl_3_/MeOH (1/1) to provide six fractions (4-1′–4-6′). The Faction 4-5′ contained the DPHC. The structure of DPHC (Figure 1A) was confirmed by comparing its NMR spectral data with those obtained from previous studies [33,34].

### 3.4. Cell Culture

RAW 264.7 cells, the murine macrophage cell line, were obtained from the American Type Culture Collection (ATCC, Rockville, MD, USA). Cells were cultured in DMEM supplement with 10% (vol/vol) FBS and 100 U/mL penicillin-streptomycin (GIBCO, Grand Island, NY, USA). The cultured cells were maintained at subconfluence under 5% CO_2_ humidified atmosphere at 37 °C.

### 3.5. Cell Viability

Cell viability was determined by EZ-CyTox (WST-1) assays (Daeil Lab Inc., Seoul, Korea). RAW264.7 cells were seeded on 96-well culture plates at a density of 1.5 × 10^5^ cells/mL and were maintained in an incubator for 18 h. The medium was then replaced with a new medium, DMEM supplemented with 10% FBS and with 100 U/mL penicillin-streptomycin. The cells were treated with LPS in the absence or presence of various DPHC concentrations. After incubation for 24 h, 100 μL of cells were removed from cells from each well. The remaining cells were treated with 5 μL of WST per well in a 5% CO_2_ atmosphere incubator for 2 h at 37 °C. The light absorbance of each well was quantified using a VersaMax ELISA microplate reader (Molecular Devices, Sunnyvale, CA, USA) at 450 nm.

### 3.6. ELISA

The 100 μL of cell culture supernatants, mouse serum or standards in a reagent diluent were added to each well of the ELISA kit well plate and incubated for 2 h. The plates were then washed, and 100 μL of the detection antibody in a reagent diluent were added to each well for 2 h. The plates were then washed again, and 100 μL of streptavidin conjugated with horseradish-peroxidase (HRP) was added for 20 min. The plates were then washed, and 100 μL of substrate solution was added to each well in a dark place for 20 min. Then, 50 μL of stop solution were added to each well. The reaction was terminated, and the optical density of each well was determined using a VersaMax ELISA microplate reader (Molecular Devices, Sunnyvale, CA, USA) at 450 nm.

### 3.7. Western Blot Analysis

Protein quantification of the supernatants was measured by the Bradford assay (Bio-Rad, Hercules, CA, USA). Aliquots of the lysates were separated on a NuPAGE 4%–12% bis-Tris gel (Invitrogen, Carlsbad, CA, USA). The proteins were transferred onto a polyvinylidene difluoride (PVDF) membrane using an iBlot gel transfer device (Invitrogen, Carlsbad, CA, USA). Then, membranes were blocked with 5% non-fat skim milk solution, and the membranes were incubated with primary antibodies at 4 °C overnight. The membranes were then washed several times with Tween 20-Tris-buffered saline (TBST) and incubated with secondary HRP-linked anti-rabbit or anti-mouse IgG, respectively, for 90 min at room temperature. After washing, immunoactive proteins were determined using the WEST-ZOL (plus) Western blot detection system (iNtRON Biotechnology, Gyeonggi, Korea).

### 3.8. Confocal Laser Scanning Microscopy Analysis

Cells were seeded onto coverslips in a 6-well plate and then fixed with 3.5% formaldehyde (PFA) in PBS for 30 min. The cells were washed with PBS, then treated with 0.1 M glycine for 15 min and permeabilized with PBS containing 0.1% Triton X-100 for 10 min. After several washings with PBS, the cells were blocked in PBS containing 3% BSA and 0.1% Triton X-100. The primary antibodies diluted by 1:200 were treated at 4 °C, overnight. The cells were then washed with Tween 20-PBS (PBST), and DyLight488 conjugated donkey anti-rabbit secondary antibodies diluted by 1:200 were applied for 30 min to the cells. This process was performed in a dark place at room temperature. After the cells were washed several times, the coverslips were mounted to slides using a VECTASHIELD mounting media with DAPI (Vector Labs, Burlingame, CA, USA). The images were visualized using an FV500 confocal microscope (Olympus, Tokyo, Japan).

### 3.9. Disease Models

To induce experimental AD, mice were sensitized by applying 1% dinitrochlorobenzene (DNCB; Tokyo Kasei Kogyo Co., Ltd., Tokyo, Japan; 150 μL) or vehicle alone to the abdomen (on Day 7). On Day 0, mice were challenged again by applying 100 μL of 0.3% DNCB to the ears on every other day for up to 30 days. From Day 12 until the completion of the experiment, the mice were painted with hydrocort cream (Green Cross, Yongin, Korea) containing 2 mg/g hydrocortisone valerate and DPHC (10 and 100 mg/kg) to the ears on every other day. The mice were sacrificed on Day 31.

### 3.10. Macroscopic Edema and Histological Evaluation

In the experimental AD mouse model, DNCB stimulation elicited ear edema, and ear thickness was measured using a digital thickness gauge (Mitutoyo, Tokyo, Japan). Ear tissues were fixed with 10% formalin and embedded in paraffin. Paraffin sections (3 μm each) were stained with H & E.

### 3.11. Statistical Analysis

Statistical differences between the groups (experimental and control groups) were analyzed by the Student’s *t*-test. The values were reported as the means and standard deviation (means ± SD) of at least three independent experiments. *p*-values <0.05, <0.01 and <0.001 were considered to be statistically significant.

**Figure 6:**
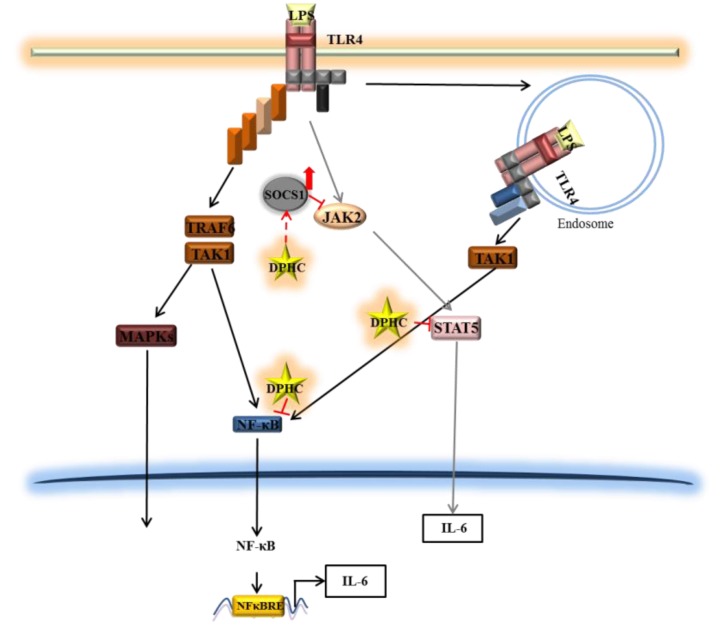
A diagram illustrating the hypothetical mode of action of DPHC on IL-6 production in LPS-stimulated RAW264.7 cells.

## 4. Conclusions

DPHC strongly inhibited the production of IL-6 in murine macrophage RAW 264.7 cells. DPHC suppressed the phosphorylation and the nuclear translocation of NF-κB, a central signaling molecule in the inflammation process induced by LPS. Additionally, DPHC inhibited the expression of STAT5 and increased that of SOCS1. Furthermore, TPCK and JI reduced the LPS-induced IL-6 production in RAW 264.7 macrophages. These findings demonstrate that DPHC inhibits IL-6 production via downregulation of the NF-κB and Jak2-STAT5 pathway and upregulation of SOCS1. Furthermore, DPHC alleviated several symptoms (ear edema, lymph node size, serum IgE level, mast cell infiltration) in experimental atopic dermatitis induced in mice. Taken together, DPHC may be a valuable anti-inflammatory compound isolated from marine plants (Figure 6).
